# Diagnosis of naturally occurring lumpy skin disease virus infection in cattle using virological, molecular, and immunohistopathological assays

**DOI:** 10.14202/vetworld.2021.2230-2237

**Published:** 2021-08-27

**Authors:** Dawlat M. Amin, Gehan Shehab, Rawhya Emran, Rabab T. Hassanien, Gehan N. Alagmy, Naglaa M. Hagag, Mervat I. I. Abd-El-Moniem, Ahmed R. Habashi, Essam M. Ibraheem, Momtaz A. Shahein

**Affiliations:** 1 Department of Pathology Research, Animal Health Research Institute, Agriculture Research Center, 12618 Dokki, Giza, Egypt; 2 Department of Virology Research, Animal Health Research Institute, Agriculture Research Center, 12618 Dokki, Giza, Egypt; 3 Department of Pathology Research, Animal Health Research Institute (Zagazig Branch), Agriculture Research Center, 12618 Dokki, Giza, Egypt; 4 Genome Research Unit, Animal Health Research Institute, Agriculture Research Center, 12618 Dokki, Giza, Egypt

**Keywords:** histopathology, immunohistochemistry, isolation, lumpy skin disease virus, polymerase chain reaction

## Abstract

**Background and Aim:**

Lumpy skin disease (LSD) is a contagious viral disease that has great economic losses among Egyptian breeding flocks. The present study was designed to compare the results of different diagnostic approaches used for the diagnosis of LSD virus (LSDV).

**Materials and Methods:**

A total of 73 skin nodule samples were collected from suspected infected cattle with LSDV from some Egyptian governorates during 2019 and 2020. Trials for virus isolation (VI) and identification on embryonated chicken eggs (ECEs) were conducted. Molecular detection, histopathological, and immunohistochemical examination were also conducted.

**Results:**

The virus was isolated into ECEs, and 58 samples of 73 were positive and gave a characteristic pock lesion on the chorioallantoic membrane. Twenty-two representative nodular skin specimens of the 58 positive samples were selected to be used for molecular, histopathological, and immunohistochemistry (IHC) diagnosis. Conventional polymerase chain reaction succeeded in detecting LSDV DNA in all tested 22 skin nodule samples. Histological examination of skins of different cases revealed various alterations depending on the stage of infection. IHC was used as a confirmatory test for detecting LSDV antigen in the tissues of the skin nodules of infected cattle using specific anti-LSDV antibodies. Lumpy skin viral antigen was detected within the cytoplasm of the epidermal basal cells layer and prickle cell and within the cytoplasm of the hair follicles’ epithelial outer and inner roots.

**Conclusion:**

This study confirmed the prevalence of LSDV infection in different Egyptian governorates during 2019 and 2020. In addition, histopathology and IHC could be potential methods to confirm Lumpy skin disease infection besidesVI and molecular detection.

## Introduction

Lumpy skin disease (LSD) is a highly contagious transboundary skin disease in cattle caused by the LSD virus (LSDV). This virus belongs to the genus *Capripoxvirus* of the *Poxviridae* family. *Capripoxvirus* genus also including goatpox virus and sheeppox virus (SPPV) that share 97% nucleotide identity and are serologically cross-protective [1]. In 1929, the first case of LSD was identified in Zambia (Rhodesia), and it was initially diagnosed as pseudo-urticaria of cattle [2]. The disease spread sporadically into Botswana by 1943 [3], and then it spread to South Africa, affecting over eight million cattle and causing significant economic loss. Since then, the disease becomes endemic in most African countries, including Egypt, which was confirmed for the first time in 1988 with recurrent occurrence in the following years till present [4-6]. In the past decade, LSD further extended to Middle Eastern, European, and West Asia regions [7]. LSD is listed as notifiable by the Office International des Epizooties (OIE) because of its rapid spread and substantial economic losses [8]. It causes reduction in milk production, loss of weight, damage to hides, sterility in bulls, and abortion of pregnant cattle. Moreover, the high cost is needed for eradication measures and vaccination programs [9]. The primary method of transmission is mechanical by arthropod vectors [10]. The severity of the clinical signs of LSD depends on the strain of capripoxvirus, age, immunological status, and breed of the host. The disease’s mortality is often <5%; however, morbidity usually reaches 20% but can vary between 3% and 85% [11,12]. The disease presents itself clinically as distinct nodular lesions on the skin and underlying tissues of infected animals. The lesions can vary widely from one animal to another; even within the same herd, recovery is slow, and often, scars are left on the hides of animals [11,13].

Diagnosis of LSD mainly depends on the typical clinical signs, differential diagnosis, and application of various diagnostic laboratory techniques for detection and confirmation of the disease, such as electron microscopy examination, virus isolation (VI), serological tests (serum neutralization test, agar gel immune diffusion, indirect enzyme-linked immunosorbent assay, and indirect fluorescent antibody technique [IFAT]), and real-time or conventional polymerase chain reaction (PCR) [7,12,14]. Viral isolation and identification as well as PCR methods are the most sensitive methods for detecting LSDV in skin samples. However, viral isolation is a gold standard for LSDV diagnosis; it is time-consuming as the protocol takes several weeks to isolate LSDV in tissue cultures or chorioallantoic membrane (CAM) of embryonated chicken egg (ECE) [15]. Immunohistochemistry (IHC) is an essential tool for diagnosing many animal diseases, including LSDV;several authors have reported it as a direct method for detecting the pathogenic antigen distribution using specific anti-LSDV antibodies in skin nodules of infected cattle [16,17].

This study was designed to assess the various laboratory diagnostic methods for naturally infected cases of LSD in cattle using viral isolation and identification, molecular, histopathological, and IHC assays.

## Materials and Methods

### Ethical approval

The study was approved by Ethics Committee on Animal Experimentation in Animal Health Research Institute (AHRI), Agriculture Research Center, Egypt (approval no 16793).

### Study period, location, and sampling

A total of 73 nodular skin biopsy samples were collected from cattle suspected clinically to be infected with LSDV during 2019 and 2020 and submitted to AHRI for laboratory diagnosis of the disease. These cattle were scattered throughout different Egyptian governorates (Menofia, Behira, Gharbia, Ismailia, Kafr El-Sheikh, Damitta, and Sharkia). The diseased animals were suffering from fever with the appearance of various stages of firm nodules distributed throughout the skin. Skin biopsies from all 73 cattle were collected aseptically through surgical excision under local anesthesia comprising the epidermis and dermis of nodular skin lesions and from the surrounding area. Each of these samples was divided into two parts: One part was maintained in 15 mL sterile screw-capped tube and transported on an icebox to AHRI for viral isolation and conventional PCR, and the other part was kept in 10% neutral formalin used for histopathology and IHC examination.

For viral isolation and PCR, the collected samples were prepared as described by Zeedan *et al*. [18]. The nodules were minced using sterile scissors and forceps and then ground with a sterile pestle in a mortar with sterile sand and an equal volume of sterile phosphate-buffered saline (PBS) containing sodium penicillin (1000 IU/mL), streptomycin (1 mg/mL), and mycostatin (100 IU/mL) to reach 10% final conc. (W\V). The homogenized suspension was freeze and thawed thrice and then partially clarified via centrifugation at 3000 rpm for 10 min, and the supernatant was stored at −80°C for further analysis.

### VI on ECEs and identification through IFAT

The trial for LSDV isolation on CAM of specific pathogen-free (SPF)-ECEs was conducted according to OIE [12]. Each sample suspension (200 μL) was inoculated in 11-13 days SPF-ECE through the CAM. In addition, sterile PBS was inoculated in SPF to be used as a negative control. The inoculated eggs were incubated for 5-7 days at 37°C with daily candling. After 5-7 days, the CAMs were carefully collected and examined for the presence of the characteristic pock lesions of LSDV. Suspected CAMs were prepared as described and further passaged in another ECE, and three serial blind successive passages were conducted. IFAT tested the positive CAMs for viral identification [12]. For this technique, reference strain and antisera were obtained from the Virology Department, AHRI, and anti-bovine IgG conjugated with fluorescent isothiocyanate used for IFAT.

### Detection of LSDV through conventional PCR

#### Viral DNA extraction

QIAamp^®^ DNA Mini Kit (QIAGEN GmbH, Germany) was used for LSDV DNA extraction from prepared 22 skin nodule representative samples following the manufacturer’s instructions.

#### PCR amplification

The PCR amplification reaction was conducted according to the manufacturer’s instructions using AmpliTaq Gold™360 Master Mix, supplied by Applied Biosystems™, Thermo Fisher Scientific, Waltham, MA, USA. The PCR primers used for amplification of partial GPCR gene that is specific for the LSDV were manufactured by Bio Basic, Canada Inc. of Markham, Ontario, Canada, with the following sequence: LSDVF (5′-AGT ACA GTT AGT AGC GCA ACC-3′) and LSDVR (5′-GGG TGA ACT ACA GCT AGG TAT C-3′) to amplify 554 bp fragment of the partial GPCR gene [19]. The PCR reaction of the total volume of 25 μL was conducted with 6 μL extracted DNA, 12.5 μL2× AmpliTaq Gold™360 Master Mix, and 1 μL 20 pmol forward and reverse primers and then completed up to a final volume with nuclease-free water. DNA amplification was conducted in BIO-RAD^®^ PCR system T100 thermocycler (Bio-Rad, Hercules, California, USA) preheated and adjusted at cycling protocol: Initial denaturation at 95°C for 10 min, then 40 cycles of denaturation at 95°C for 55 s, annealing at 50°C for 55 s, and extension at 72°C for 1. 5 min, followed by a final extension at 72°C 10 min. Reference LSDV strain was used as a positive control and SPPV as a negative control. The amplified PCR products were analyzed by 1. 5% agarose gel electrophoresis. The DNA band of the predicted size (554 bp) was visualized and detected using Molecular Imager Gel Doc XR+ Imaging system (Bio-Rad) using Image lab™ software (https://image-lab-4-0.software.informer.com/) for analysis of gel images compared with 100 bp DNA molecular weight marker.

### Histopathological examination

Twenty-two representative skin nodule specimens fixed in 10% neutral formalin were used for histopathological examination; the tissues were dehydrated through graded alcohols and embedded in paraffin wax; 4-μm-thick serial sections were cut, stained with hematoxylin and eosin, and examined using a light microscope equipped with an ocular micrometer (Nikon Eclipse E600, Japan) [20].

### IHC

IHC was used as a confirmatory test for detecting LSDV antigen distribution in the tissues of the skin nodules of infected cattle using specific anti-LSDV antibodies. Deparaffinized skin tissues (5-7 μm ­section) were treated with avidin-biotin-peroxidase complex (ABC, SNF Medical). The antibody against LSDV used as a primary antibody, was obtained from the Veterinary Serum and Vaccine Research Institute, Abbassia. The technique was conducted according to the manufacturer’s instructions.

## Results

### Clinical signs

**Figure-1:**
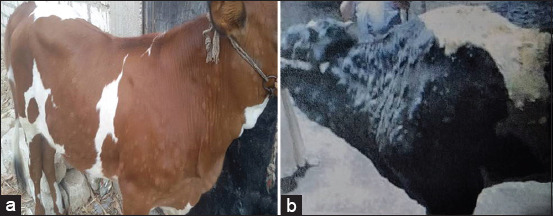
(a and b) Various stages of firm skin nodules distributed through the animal’s body.

Infected cattle (2-4 years old) showed fever and various stages of firm nodules within the skin associated with generalized lymph node enlargement. Although the skin nodules vary in numbers from a few to a hundred distributed throughout the animal’s body (Figures-1a and b), many of these nodules were ulcerated. The central areas of some nodules appeared indurated; other nodules appeared with necrotic centers or suppurated. The nodules involve the dermis, epidermis, adjacent subcutis, and musculature, which were painful on palpation.

### VI on ECE and identification via IFAT

**Figure-2:**
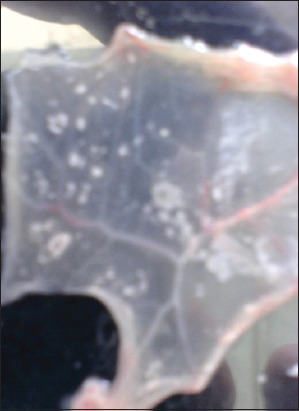
Characteristic pinpoint to pinheaded pock lesions of examined samples for suspected lumpy skin disease on chorioallantoic membrane of specific pathogen-freeembryonated chicken eggs after three blind passages.

**Figure-3:**
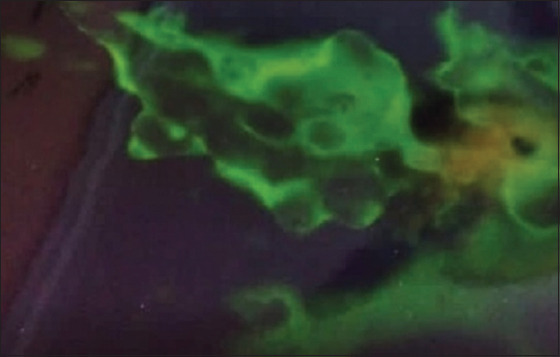
Chorioallantoic membrane of embryonated chicken eggs showing specific apple green fluorescence emission as a positive result for local lumpy skin disease virus.

Out of 73 inoculated samples in ECE, 58 samples (79.45%) gave characteristic pock lesions on CAMs. The pock lesions appeared opaque, white, circular, pinpoint to pinhead in size, scattered at the inoculation site with a slight thickening and edema of the membrane and congestion of blood vessels and appeared more prominent at the third passage (Figure-2). The other 15 samples did not show any changes, and no pock lesions have been observed on the examined CAMs. Consequently, these 15 samples were considered negative samples. Positive CAMs were confirmed through IFAT and appeared as diffuse membranous with apple green fluorescence emission using specific antisera for LSDV (Figure-3).

### Detection of LSDV through conventional PCR

**Figure-4:**
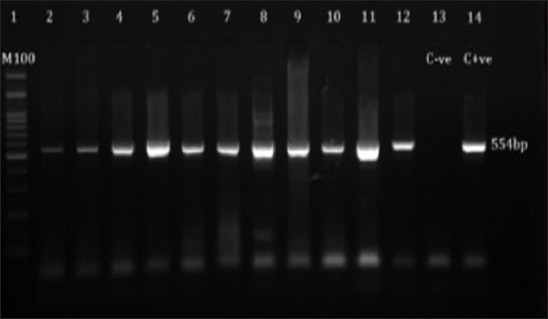
Agarose-gel electrophoresis of amplified products of 554 bpin size obtained from lumpy skin disease virus(LSD) DNA using LSD- specific primers of GPCR gene. Lane 1 (100bpDNA size marker); lane 13 (negative control); lane (2-12) (LSD virus positive samples); lane 14 (positive control).

PCR succeeded in identifying LSDV DNA in the 22 examined samples and gave specific bands at 554 bp (Figure-4).

### Histopathological examination

**Figure-5:**
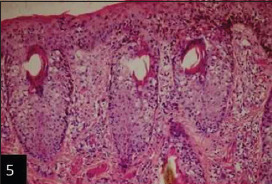
Skin showing epidermal rete ridge formation (hematoxylin and eosin [H&E] 100×),

**Figure-6:**
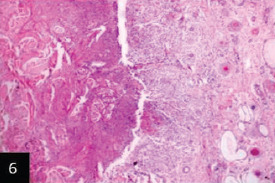
Skin showing epithelial sequestration; separation and granulation tissues formation replacing the epidermal cell layer leaving deep ulcer these are associated with marked thrombosis of dermal blood and lymphatic vessels (H&E 100×),

**Figure-7:**
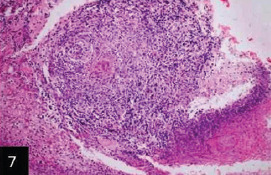
Skin showing granulomatous tissues filling the ulcerated areas (H&E stain 200×),

**Figure-8:**
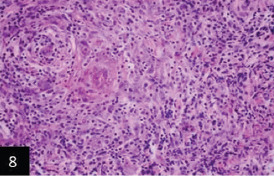
Higher magnification for Figure-7 that showing that the granulomatous tissues filling the ulcer consisting collection of macrophages; epithelioid cells; lymphocytes and plasma cells (H&E 400×),

**Figure-9:**
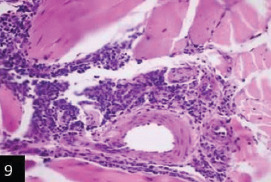
Skin showing the same granulomatous reaction around the blood vessels on deep dermal muscle layer (H&E stain 200×),

**Figure-10:**
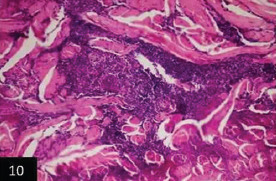
Skin showing granulomatous reactions in between deep dermal muscles and around dermal vasculature (H&E 400×).

**Figure-11:**
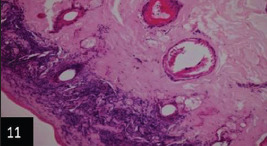
Skin showing fibrous tissue proliferating the dermal layer associated with thrombosis of dermal vasculatures (200×),

**Figure-12:**
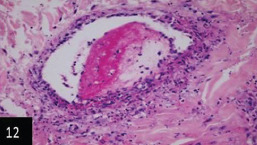
Skin showing marked vasculitis and thrombosis of dermal artery the invading cells is round one (400×),

Histological examination of the skin of different cases revealed various alterations. Many cases showed a proliferation of epidermis with rete ridge formation, and this was associated with marked hydropic degeneration of the epidermal cell layers (Figure-5); other cases showed significant necrosis of the epidermal cell layers proceeding to epidermal separation, leaving deep ulceration. In the majority of cases, these deep ulcers appeared filled with granulation tissues (Figure-6). In other cases, granulomatous reaction was seen in and around this ulceration. These granulomatous tissues consist of plasma cells, macrophages, lymphocytes, epithelioid cells, and fibrocytes (Figures-7 and 8). The same granulomatous reactions were seen around the blood vessels on dermal layers and in-between dermal muscles (Figures-9 and 10). Subepidermal fibrous tissue proliferation was seen in some cases, accompanied by dystrophic calcification throughout the dermal layer. These histopathological changes were associated with vasculitis, lymphangitis, and thrombosis of dermal blood and lymphatic vasculatures. Moreover, marked damages to the endothelial cells of the dermal blood vessels caused variable degrees of thrombosis and vasculitis (Figures-11 and 12). The presence of various homogenous accompanied the previous histopathological changes, eosinophilic intracytoplasmic inclusion bodies within the epithelial cells of the prickle cell layer (Figures-13a and b), sebaceous glands, hair follicles (Figures-14 and 15), and within the macrophages infiltrating the subepidermal layer (Figures-16a and b).

**Figure-13a):**
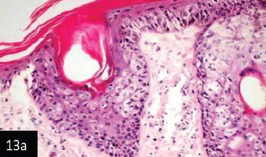
Skin showing hyperplasia of epidermal cell layer forming dawn growth of rete ridge and these are associated with intra-cytoplasmic inclusion bodies (200×),

**Figure-13b):**
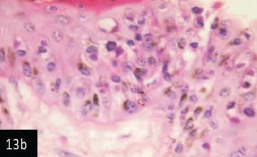
Skin showing acanthosis and proliferation of the epidermis associated with intracytoplasmic inclusion bodies (H&E stain 400×),

**Figure-14:**
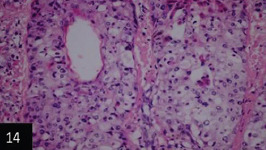
Skin showing eosinophilic homogenous intracytoplasmic inclusion bodies within the epithelia of sebaceousglands (H&E stain 200×),

**Figure-15:**
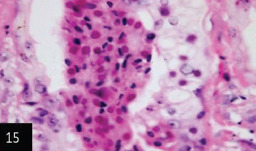
Skin showing numerous numbers of homogenous eosinophilic intracytoplasmic inclusion bodies within epithelial cells of sebaceous glands (H&E stain 400×),

**Figure-16:**
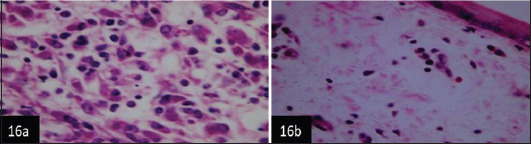
(a and b) Skin showing numerous intracytoplasmic inclusion bodies within the macrophages infiltrating the dermal layer (H&E 1000×).

### IHC

**Figure-17:**
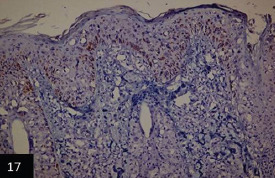
Skin showing: Specific immunoperoxidase of lumpy viral antigen within different epidermal cells layers and within that of hair follicles *counterstained with Mayer’s Hematoxylin (100×),

**Figure-18:**
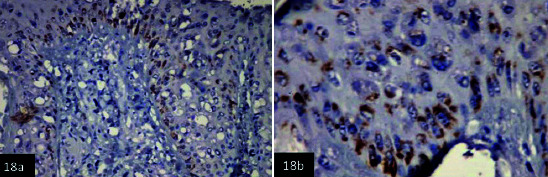
(a and b) Skin showing specific granular golden brown immunoperoxidase staining of lumpy viral antigen within different cells layer of the epidermis. *counterstained with Mayer’s hematoxylin (a 200×) (b 400×),

**Figure-19:**
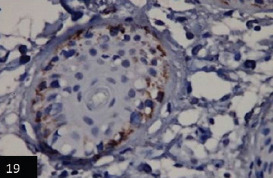
Skin showing specific immunoperoxidase reaction against lumpy viral antigen within the epithelia cells lining the hair follicles*counterstained with Mayer’s hematoxylin (400×).

IHC investigations revealed the detection of lumpy skin viral antigen within the cytoplasm of epidermal basal cells layer and prickle cell; the reactions appeared as a granular golden brown immunoperoxidase staining of viral antigen (Figures-17,18a and b). Furthermore, a specific immunoperoxidase reaction against LSD antigen was detected within the cytoplasm of the epithelial outer and inner root of hair follicles (Figure-19).

## Discussion

LSD is a poxvirus that causes systemic disease in cattle [6,16]. Diagnosis of LSDV depends mainly on the clinical signs; however, mild and unapparent disease may be difficult to diagnose, and rapid laboratory methods are needed to confirm the diagnosis [21]. The clinical diagnosis of LSD is not difficult for those familiar with the disease, but those who are not quite experienced can readily confuse lesions with many other conditions [22]. For example, Urticaria, insect and tick bites, or insect stings may develop lesions similar to LSD clinically, but the absence of eosinophils and the presence of a deep vasculitis should rule these conditions out. Herpesvirus infection can be differentiated histologically from LSD by examining early lesions for typical eosinophilic intranuclear inclusion bodies. Cutaneous lymphosarcoma, streptothricosis, and tuberculosis can also be differentiated histologically [22,23].

The present study was conducted on 73 skin nodules samples collected in 2019 and 2020 from different governorates of Egypt. The infected cattle showed typical lesions for LSDV infection characterized by fever and observation of few or many skin nodules and enlarged peripheral lymph nodes. Those clinical signs were comparable with other previous descriptions [5,24,25]. Furthermore, the present work showed that the disease was most prevalent in the age group between 2 and 4 years, and this came in agreement with Gharban *et al*. [26], who attributed the lowest infection attack among cattle less than 2 years because of passive maternal immunity that protects calves from LSDV infection.

Early observation of clinical signs of LSDV, followed by rapid laboratory diagnostic confirmation of the suspected disease, is a vital step for LSDV control. In this study, all collected samples were passaged on CAM of ECE. The obtained results showed that 58/73 (79.45%) gave characteristic pock lesions for LSDV. These findings matched well with the results of Tamam [27] and Zeedan *et al*. [18]. IFAT further identified the positive CAMs. This technique serves as a rapid, effective, and economical method for laboratory confirmation of LSDV. Depending on the ECE for isolation and identification of the virus may solve the problem of viral isolation on tissue culture cells, especially that the virus has a narrow range of susceptible cell cultures. Besides the fact that capripox VI is difficult, it grows slowly and requires additional passages, even if cultured in the most sensitive primary cell cultures [15].

The molecular investigation in this study was used to confirm the presence of LSDV DNA in 22 skin nodule samples. The selection of this number was representative and not collective because of the high cost of conventional PCR assay. Moreover, the same number of samples was used to make pathology and histochemistry comparisons. The used conventional PCR assay showed high specificity and sensitivity as the test gave positive results with all tested samples with no cross-reaction. The conventional PCR result was fully correlated with field diagnosis on the basis of clinical symptoms and completely matched the VI, pathology, and histopathology results. Thus, there was no need to test more samples. The detection of LSDV via PCR is considered an accurate and rapid confirmatory test, as results were obtained within 24 h after sample collection, whereas VI takes several weeks and may require several passages [28]. By contrast, previous studies supported using real-time PCR assay as it offers more advantages. For example, it is a quantitative test, a more sensitive, simpler, and faster diagnosisof LSDV than conventional PCR. In addition to this, the test is used as a screening assay for the universal detection of LSDV DNA [18,29,30].

The variation in the histological findings of LSD among infected cattle has also been described by Tuppurainen *et al*. [21]. Similar results to our findings were previously observed by Gharban *et al*. [26], who reported that replication of the virus is accompanied by the formation of intracytoplasmic inclusion bodies in skin lesions of infected cattle. Furthermore, Prozesky and Bernard [31] concluded that vasculitis and thrombosis leading to edema and necrosis. Moreover, Awadin *et al*. [24] and El-Neweshy *et al*. [16] detected vasculitis affecting the small venues, capillaries, and arterioles of the deep dermis with resultant pannicular infarction. The authors suggested that the vascular effect may return to the release of cytokines by inflammatory cells (immune-mediated vasculopathy), not because of the direct endothelial injury by infectious agents. Although the pathogenesis and exact etiology of immune-mediated vasculitis is unknown, advances in molecular research have revealed that an imbalance in inflammatory cytokines is central to the pathogenesis of this condition [32]. Our results were also supported by Abdulqa *et al*. [33], who reported that the virus might infect different types of cells, including pericytes, fibroblasts, epithelial, and endothelial cells. Infected areas developed extreme vasculitis and lymphangitis because of viral replication in pericytes, endothelial cells, and possibly some cells in blood vessels and lymph vessel walls. In severe cases, an infarction can occur [31]. Cutaneous lesions may heal quickly, or they may in durate and become hard lumps, or become sequestered, leaving deep ulcers partially filled with granulation tissue, which often suppurates [33-35].

In this study, IHC was used to confirm the detection of LSD viral antigen in the skin of different cases by applying the avidin-biotin complex immunoperoxidase technique. In this investigation, demonstration of LSD viral antigen within the cytoplasm of epidermal basal cells layer and prickle cells, also in the cytoplasm of epithelial cells lining, the outer and inner roots of hair follicles were noticed. These findings are comparable with the results of several authors [12,16,36]. In this respect, Coetzer and Tuppurianen [34] reported that immunohistochemical methods, such as immunoperoxidase staining of tissue sections, can demonstrate the LSD viral antigen in acute and chronic skin lesions. Furthermore, Babiuk *et al*. [36] found that IHC of hair follicle epithelium from LSD cattle skin showing positive staining for capripox virus antigen and positive interstitial macrophages. In addition, Awadin *et al*. [24] demonstrated in skin nodules of LSD cases a positive IHC reaction inside epidermal cells and macrophages infiltrating the dermis; however, the reaction was slightly more profound in the acute stage skin nodules compared with the subacute and chronic stages. The present work of immunohistopathological studies on skin tissue nodules indicated the importance of this technique for confirmation of LSD infection, which was comparable with viral identification and PCR-positive results. Hence, this study ultimately agreed with Goswami *et al*. [37], who emphasized the importance of IHC as one of the best tools used for disease diagnosis as it provides the most direct method for identifying both the cellular and sub-cellular distribution of pathogens or antigen protein with the use of specific antibodies.

The application of laboratory diagnostic protocol for confirmation of a suspected infection is a prerequisite in any epidemic disease control strategy, particularly regarding the prevalence of the LSDV in a livestock population in the Egyptian field. The performance of various assays in this work confirmed the prevalence of LSDV infection among cattle from different Egyptian governorates during 2019 and 2020. Although all applied tests (VI and identification, PCR, histopathology, and IHC) in this work were sensitive and gave definitive diagnosis for detecting LSDV in skin nodular tissues of cattle, some techniques might not be easy to be applied in some laboratories as many laboratories lack biosafety cabinet level 2 facilities, which is essential for viral isolation and molecular assays. In such cases, we support using histopathology and IHC as essential tools for LSDV diagnosis.

## Conclusion

Conclusively, PCR stands as the most rapid and accurate method to confirm the LSD infection if laboratory facilities are available; however, histopathology and IHC can also be used in routine pathology laboratories for detecting LSDV antigen in skin nodular tissues for confirmation of LSD infection, which was comparable with the findings of virus identification and PCR results.

## Authors’ Contributions

MAS: Designed the study, GNA: Collected the samples. ARH: Collected the data of the samples. DMA, GS, RE, and EMI: Performed the pathology and histopathology for the collected samples. RTH and MIIA: Carried out the samples preparation, viral isolation, and identification. NMH: Performed the conventional PCR assay. MAS, EMI, and ARH: Made the final revision. All authors wrote the original draft, discussed the result and contributed to the final manuscript. All authors read and approved the final manuscript.
